# Clinico Socio-Demographic Profile of Children with Autism Spectrum Disorder from a Mental Health Clinic in Nepal: An Observational Study

**DOI:** 10.31729/jnma.8937

**Published:** 2025-04-30

**Authors:** Utkarsh Karki, Amit Jha, Samjhana Parajuli, Anil Sharma, Bhupendra Gurung, Dipesh Bhattarai

**Affiliations:** 1 Mental Health Unit, Synergy Mind Clinic, Kathmandu, Nepal; 2 Rainbow Center, Human Resource Training Center, Pokhara, Nepal

**Keywords:** *autism spectrum disorder*, *children*, *clinical profile*, *Nepal*

## Abstract

**Introduction:**

Autism Spectrum Disorder (ASD) is a neurodevelopmental disorder with symptoms manifesting in early childhood. There is limited information regarding the clinical scenario of ASD within Nepal. The study aims to determine the clinical and socio-demographic profile in children with ASD.

**Methods:**

This was an observational cross-section study which examined the records of children diagnosed with ASD at a Mental Health Clinic in Nepal. Approval for the study was granted by NHRC. The analysis focused on data extracted from clinic records of ASD patients spanning from 16 September 2022 to 15 March 2024 encompassing one and a half years. Data were entered and analyzed using SPSS Statistics for Windows, version 16.0 (SPSS Inc., Chicago, Ill., USA).

**Results:**

A total of 138 children were diagnosed with ASD, with a mean age of diagnosis at 42.94 ±17.49 months. Among the participants, there were 110 (79.69%) male, 105 (76.01%) first born with a male to female ratio of 3.9:1 and 114 (82.59% ) were going to regular school. Parents’ 39 (28.29%) and relatives’ 25 (18.01%) concerns were the primary reasons for seeking a diagnosis followed by referrals from pediatricians 19 (13.79%). The mean Childhood Autism Rating Scale (CARS) score of 34.33±3.99 was indicative of mild to moderate symptoms of ASD. The mean Vineland Social Maturity Scale (VSMS) score of 69.02±13.25 was indicative of mild impairment in socio-adaptive functioning.

**Conclusions:**

The study shows higher rate of ASD among males and first-born children. Early age at diagnosis is encouraging and is associated with better outcomes.

## INTRODUCTION

Autism Spectrum Disorder (ASD) is a neruodevelopmental disorder (NDD) characterized by persistent deficits in social interaction and communication and restricted, repetitive patterns of behavior, interests, or activities.^1^ The average prevalence rate of ASD in Asia is 1.48 per 1000.^2^ Recently, there has been an increasing worldwide prevalence of ASD, with an estimate of about 0.5%-1%^3^and ASD is considered an issue of public health importance.

While there is a significant amount of research on ASD in various countries, limited data are available regarding ASD in Nepal. A study by Shrestha et al. examined the age of diagnosis of ASD in Nepal, highlighting the scarcity of comprehensive data on the disorder within the country.^4^ Despite the rising prevalence of ASD, we have very few studies showing the basic but very essential components of the clinical and socio-demographic profile of children with ASD in our country. For instance, a 2024 study investigated early behavioural signs among Nepalese children with ASD, underscoring the limited scope of existing research.^5^

The study aimed to determine the clinical and socio-demographic profile of children with ASD presenting to Synergy Mind Clinic (SMC), Kathmandu, Nepal.

## METHODS

An observational cross-section study was conducted with children diagnosed with ASD between 16 September 2022 to 15 March 2024, encompassing 18 months. The study was conducted at Synergy Mind Clinic (SMC), Chandol, Kathmandu, Nepal. SMC is a private mental health clinic that especially provides dedicated services for children and adolescents with neurodevelopmental disorders and mental health problems with child and adolescent psychiatrists, psychiatrists and clinical psychologists.. With limited professionals working in this area and high demand for children with ASD the clinic receives a large number of consultation. In addition to the diagnostic evaluation the clinic also provides early intervention, parent training and therapies for children and families with ASD. The multidisciplinary team comprises of a) child and adolescent psychiatrists b) clinical psychologists c) physiotherapist/sensory integration trained therapist d) speech and language pathologist and e) certified parent child intervention trainer. Child and Adolescent Psychiatrist or Psychiatrist first evaluates the child and family which is followed by another evaluation by a clinical psychologist. Sensory integration trained therapist, speech and language pathologist and certified parent child intervention trainer are involved in providing therapy services to autistic children and their family members.

Ethical approval was obtained from Nepal Health Research Council (NHRC) (Reference Number: 1870). All diagnosed cases of ASD based on detailed evaluation by a Child and Adolescent Psychiatrist and Clinical Psychologist in the records of the clinic for 18 months were included in the study. All cases had undergone a detailed clinical evaluation based on unstructured psychiatric interviews and observation with both the child and parent/s by a child and adolescent psychiatrist followed by a clinical psychologist. Childhood Autism Rating Scale (2^nd^ Edition Revised) was used to diagnose and measure the severity of ASD and Vineland Social Maturity Scale (VSMS) was used to analyse the socio adaptive functioning of children. Case records had the following information recorded: name, age, gender, birth order,address, source of referral, age of parents, type of family, family history of neruodevelopmental disorder, screen time, birth history, pre natal/perinatal/postnatal events, CARS score and VSMS score. Consent was not applicable as this was retrospective study; however, confidentiality of data was maintained as patients and their family members were not recruited directly for the study. Data were entered and analyzed using SPSS Statistics for Windows, version 16.0 (SPSS Inc., Chicago, Ill., USA). Descriptive statistics were used to calculate frequencies, percentage and mean.

## RESULTS

Shapiro-Wilk test and Kolmogorov-Smirnov tests normality revealed that the continuous variables; CARS score, VSMS score, and age were found to be normally distributed.

The mean age of the study population was found to be 42.94±17.49 months, father's age was 32.3±.22 years and that of mother was 29.06±4.50 years. A total of 110 (79.69%)of the participants were male and 83 (60.01%) werefrom Bagmati province. About the educational status, 114 (82.59%) participants were attending regular school. Among the participants 75 (54.29%) belonged to nuclear families, 105 (76.01%) were the first-born child, and 129 (93.49%) had no family history of neurodevelopmental disorders (NDD), (Table 1).

**Table 1:** Clinical profile of study population (n=138).

Socio-demographic Variables	n (%)
**Gender**	
Male	110 (79.69)
Female	28 (20.29)
**Province**	
Koshi	16 (11.59)
Madesh	15 (10.89)
Bagmati	83 (60.01)
Gandaki	3 (2.19)
Lumbini	15 (10.89)
Karnali	1 (0.69)
Sudurpaschim	5 (3.59)
**Schooling**	
Regular school	114 (82.59)
Special school	5 (3.59)
Not Going to School	19 (13.79)
**Family Type**	
Nuclear	75 (54.29)
Joint	42 (30.39)
Extended	21 (15.19)
**Birth Order**	
First	105 (76.01)
Second	30 (21.69)
Third	1 (0.69)
Fourth	2 (1.39)

The primary reason for the visit was due to concern by parents and relatives in 64 (46.39%) cases, followed by referrals from pediatricians in 19 (13.79%) cases and information obtained through social media in 15 (10.89%) cases. Regarding birth history, 98 (71%) participants were delivered via lower segment cesarean section (LSCS). A total of 131 (94.89%) were born at term, 123 (89.01%) were Appropriate for Gestational Age (AGA), 135 (97.83%) had an immediate cry at birth, and 117 (84.79%) had no any perinatal complications. The mean CARS score was 34.33±3.99 and belonged to the category of mild to moderate symptoms of ASD (N=89, 64.49%). The mean VSMS score was 69.02±13.25 (Table 2).

**Table 2:** Clinical profile of study population (n=138).

Clinical variable	N (%)
Source of referral
Self	39 (28.29)
Pediatrician	19 (13.79)
ENT surgeon	6 (4.29)
Speech Therapist	1 (0.69)
Relatives	25 (18.01)
Social Media	15 (10.89)
School	7 (5.01)
Psychiatrist	7 (5.01)
Autism centers	12 (8.69)
Physiotherapist	6 (4.29)
Physician	1 (0.69)
Family history of NDD
Yes	9 (6.49)
No	129 (93.49)
Screen Time
Yes	138 (100)
No	-
Delivery
SVD	38 (27.49)
Assisted	2 (1.39)
LSCS	98 (71)
Gestational Age
Term	131 (94.89)
Pre-term	7 (5.01)
Birth Weight
LBW	12 (8.69)
AGA	123 (89.01)
LGA	3 (2.19)
Birth Cry
Yes	135 (97.79)
No	3 (2.19)
**Prenatal history**	
GDM	9 (6.49)
HTN	3 (2.19)
Hypo-hyroidism	7 (5.01)
Nill	117 (84.79)
GDM + HTN	1 (0.69)
Anxiety Disorder	1 (0.69)
**Perinatal hypoxia**	
Yes	3 (2.19)
No	135 (97.79)
**Postnatal**	
NICU Admission	17 (12.29)
No NICU Admission	121 (87.69)
**CARS Severity**	
No to minimal	14 (10.01)
Mild to moderate	89 (64.49)
Severe	35 (25.39)

SVD: Spontaneous Vaginal Delivery; LSCS: Lower Segment Caesarean Section; LBW: Low Birth Weight; AGA: Appropriate for Gestational Age; LGA: Large for Gestational Age; GDM: Gestational Diabetes Mellitus; HTN: Hypertension; NICU: Neonatal Intensive Care Unit; CARS: Childhood Autism Rating Scale; VSMS: Vineland Social Maturity Scale; NDD: Neurodevelopmental Disorders.

## DISCUSSION

In our study there was 138 children with newly diagnosed ASD. The mean age of diagnosis (AoD) in this population was 42.94 (SD: ±17.49 months). A study done in Nepal on 2015 found 58 months as the mean AoD in children with ASD.“Earlier AoD in our study could be due to increase in awareness in the community and professionals, increase in the availability of child and adolescent mental health services and number of professionals providing services to this population. Early diagnosis is essential for early intervention, which can significantly improve developmental outcomes.^6^ The study found a male predominance 110 (79.69%), which aligns with the global trend where ASD is more frequently diagnosed in males than females.^6^ From our study, male to female ratio was found to be 3.9:1 which is comparable with western literature which shows the male to female ratio of 4:1.^7^ A significant proportion of participants were from the Bagmati province which could be attributed to the location of the clinic in Kathmandu, the capital city, which has better healthcare facilities and greater awareness of mental health problems compared to other regions.

The mean age of fathers and mothers age at conception was 32.3 (SD: ±5.22) and 29.06 (SD: ±4.50) years respectively. Parental age has been studied as a potential risk factor for ASD, with some studies suggesting a higher risk associated with older parental age.^8^ Most children in the study belonged to nuclear families and were first-born. The predominance of nuclear families is the reflection of broader social changes in family structures in urban Nepal. Firstborn children being more frequently diagnosed with ASD could be due to heightened parental concern and vigilance with their first child, leading to earlier detection and intervention.^9^

Parents’ and relatives’ concerns were the primary reasons for seeking a diagnosis followed by referrals from pediatricians and information from social media. This underscores the crucial role of family awareness in the early detection of ASD. The impact of social media highlights the increasing accessibility of information and the importance of digital literacy in health awareness. The majority of children were delivered via lower segment caesarean section (LSCS), were born at term with no perinatal complications. Delivery via LSCS has been associated with a higher risk of Autism Spectrum Disorder (ASD) in children. Recent studies indicate a potential link between LSCS delivery and increased ASD risk, although the exact mechanisms remain unclear.^8^

The mean Childhood Autism Rating Scale (CARS) score of 34.33 (SD:±3.99)was indicative of mild to moderate symptoms of ASD. The mean Vineland Social Maturity Scale (VSMS) score of 69.02(SD:±13.25) was indicative of mild impairment in socio-adaptive functioning. Existing literature highlights the impact of ASD severity on adaptive functioning.^10^

Children attending school had significantly higher VSMS scores, suggesting that school attendance positively impacts social maturity and adaptive functioning. School environments provide structured social interactions and learning opportunities essential for children with ASD.^11^ First-born children had higher VSMS scores, possibly due to receiving more parental attention and resources, which can enhance developmental outcomes^12^were significant differences in VSMS scores based on the mode of delivery, with children born via LSCS showing lower scores. This finding is intriguing and warrants further research to understand the underlying factors, which could include the impact of birth complications or early life stressors associated with surgical deliveries.^13^

The major limitation of the study is the retrospective design. The study is also limited by its sample size and the focus on a single clinic, which may not represent the broader population. Additionally, the reliance on parental reports and clinical referrals could introduce selection bias. Despite all the drawbacks, it is a comprehensive review of all the available sociodemographic and clinical data on ASD in children.

ASD not only affects the child and the family but also has direct and indirect cost implications on the nation as resources have to be utilized in providing health care, support for education, and rehabilitative services for thesechildren.^14, 15^

The findings of this study have several implications for clinical practice and policy-making in Nepal: a) early diagnosis and Intervention: The young mean age of diagnosis highlights the importance of early screening and intervention programs. Policies should promote routine developmental screenings in pediatric care settings to facilitate early identification and support. b) family education and support: Given the significant role of parental concern in seeking a diagnosis, educational programs for parents and families about the signs and symptoms of ASD and the importance of early intervention are crucial. c) school inclusion programs: The positive impact of school attendance on adaptive functioning underscores the need for inclusive education policies that support children with ASD in mainstream schools. Training for teachers and school staff on ASD can help create supportive learning environments.

## CONCLUSIONS

Our study shows higher rate of ASD among males and first-born children, with most of them attending regular schools. Early age at diagnosis and parents’ and relatives’ concerns being the primary reasons for seeking a diagnosis is encouraging and is associated with better outcomes.
